# Draft Genome Sequences of 77 Endemic Multidrug-Resistant Mycobacterium tuberculosis Strains of SIT41 (TUR) Spoligotype from Bulgaria

**DOI:** 10.1128/mra.01111-21

**Published:** 2022-02-10

**Authors:** Stefan Panaiotov, Vladimir Tolchkov, Yordan Hodzhev, Borislava Tsafarova, Alberto Trovato, Daniela Cirillo

**Affiliations:** a National Center of Infectious and Parasitic Diseases, Sofia, Bulgaria; b WHO Collaborating Centre and TB Supranational Reference Laboratory, San Raffaele Scientific Institute, Milano, Italy; University of Maryland School of Medicine

## Abstract

Sequences of multidrug-resistant (MDR) Mycobacterium tuberculosis strains are of particular interest to study the molecular mechanisms of drug resistance evolution. Here, we report the draft genome sequences of 77 endemic multidrug-resistant Mycobacterium tuberculosis strains of SIT41 (TUR) spoligotype from Bulgaria. SIT41 spoligotype is dominant (>40%) among the MDR-TB strains in Bulgaria.

## ANNOUNCEMENT

The current control of tuberculosis mainly depends on multidrug resistance management. Multidrug-resistant M. tuberculosis (MDR-TB) strains of SIT41 (TUR) spoligotype are endemic to Bulgaria (1–4). SIT41 spoligotype is prevalent (>40%) among patients with MDR tuberculosis and a marker for MDR-TB in Bulgaria.

Seventy-seven MDR-TB strains of SIT41 (TUR) spoligotype were collected in Bulgaria from 2009 to 2019. All strains originated from Bulgarian patients with pulmonary tuberculosis. One isolate per patient was tested. The Bactec MGIT automated mycobacterial detection system (BD, USA) was applied for isolation and drug susceptibility testing at the National Reference Laboratory for Tuberculosis at the National Center of Infectious and Parasitic Diseases, Sofia, Bulgaria. All strains were rifampicin and isoniazid resistant. Genotyping of the isolated MDR-TB strains was performed, applying spoligotyping (5) and 24-loci MIRU-VNTR typing (6).

For DNA isolation, we used a fresh subculture of the isolated MDR-TB strains grown on Löwenstein-Jensen medium for 35 days at 37°C. The DNA was isolated according to the procedure described by van Soolingen (7) including incubations with lysozyme, 10% SDS, proteinase K, 10% cetyltrimethylammonium bromide (CTAB), protein/lipid extraction with phenol/chlorophorm, and DNA precipitation with isopropanol.

Before sequencing, DNAs were quantified by SYBR Green assay (Thermo Fisher Scientific, USA). Quality was validated after agarose gel electrophoresis. The libraries were prepared by using the Nextera XT DNA Library Preparation Kit (Illumina, USA) following the manufacturer’s protocol to achieve equimolar pools of each library sample. DNAs were fragmented and ligated to Illumina adapters by “tagmentation.” After the amplification step, size selection was performed with AMPure XP Beads (Beckman Coulter, USA). The size distribution of fragments was checked using an Agilent DNA High Sensitivity chip (2100 Bionanalyzer, Agilent, USA). The libraries were sequenced on an Illumina HiSeq 2500 platform (Illumina, USA) run in paired-end reads in 2 × 100 bp using an Illumina Reagent Kit V2. The sequences were attributed to each index (8 bases) without considering any mismatches. Results demonstrated that the mean number of reads was 3.2 million and mean depth of coverage was 86×, as calculated on an estimated genome size of 4.5 Mb for M. tuberculosis. Quality assessment was performed with the use of the Prinseq-lite program (v.0.20.4) (http://prinseq.sourceforge.net) (8). Sequences from Illumina sequencing were joined using the FLASH program (v.1.2.11) (9). Illumina reads were loaded into Geneious Prime software (v.2021.2.2) (10) and trimmed with the BBDuk plugin (v.1.0, https://sourceforge.net/projects/bbmap/). Adapters on the right end and the low-quality ends (quality below 20%) were trimmed, while reads shorter than 200 bp were discarded. Then, the reads were subjected to preprocessing (https://www.geneious.com/tutorials/map-to-reference/). The genomes were assembled by using the “Map to reference” tool. As a reference genome, M. tuberculosis H37Rv (GenBank accession no. NC_000962.3) was used. A consensus sequence from aligned reads was extracted. We visually confirmed the circular genomes of the M. tuberculosis strains by assessing the reads spanning the junction between the two linearized ends and overlapping with them. Annotation was generated with the NCBI Prokaryotic Genome Annotation Pipeline (PGAP) (v.4.13) (11).

### Data availability.

The Illumina raw sequencing reads and draft genomes of the 77 endemic multidrug-resistant M. tuberculosis strains of SIT41 (TUR) spoligotype from Bulgaria were deposited in GenBank under BioProject accession number PRJNA765918, SRA study number SRP339647, and BioSample accession numbers ranging between CP086331 to CP085542 and CP085617 (Table 1).

**TABLE 1:** Descriptions and GenBank accession numbers of sequenced MDR Mycobacterium tuberculosis SIT41 (TUR) spoligotype strains from Bulgaria

No.	M. tuberculosis strain/yr of isolation	Genome size (bp)	GC content (%)	No. of contigs (scaffolds)	N50	No. of CDS^*a*^	No. of reads	Coverage (×)	Accession no.	
									GenBank	SRA
1	6/2009	4,418,129	65.9	133 (1)	83,741	3,997	1,643,190	37.6	CP085613	SRR16149118
2	8/2009	4,417,820	65.5	110 (1)	70,552	4,028	1,260,834	28.80	CP085611	SRR16149122
3	11/2009	4,417,016	65.5	107 (1)	64,046	3,995	1,224,130	28.0	CP085608	SRR16149124
4	18/2009	4,418,290	65.0	130 (1)	96,487	4,021	1,241,934	28.40	CP085604	SRR16149129
5	19/2009	4,418,358	65.5	118 (1)	74,542	4,057	1,435,026	32.8	CP085602	SRR16149131
6	20/2009	4,423,816	65.8	110 (1)	89,973	4,050	2,706,862	61.8	CP085600	SRR16149133
7	22/2009	4,419,048	65.3	115 (1)	78,954	4,041	1,579,668	36.1	CP085597	SRR16149137
8	34/2009	4,418,147	65.5	101 (1)	78,892	4,049	1,548,468	35.4	CP085593	SRR16149140
9	37/2009	4,417,302	65.4	102 (1)	81,977	3,944	1,549,428	35.4	CP085592	SRR16149141
10	1/2010	4,421,044	65.1	119 (1)	66,651	3,997	1,785,090	40.1	CP085614	SRR16149117
11	7/2010	4,417,102	65.0	120 (1)	64,647	4,043	1,662,824	30.0	CP085612	SRR16149119
12	8/2010	4,416,514	65.0	143 (1)	78,919	4,036	1,585,744	36.2	CP085610	SRR16149120
13	10/2010	4,426,983	65.1	122 (1)	83,883	4,069	3,698,326	84.4	CP085609	SRR16149123
14	12/2010	4,416,642	65.1	115 (1)	63,851	4,047	1,554,696	35.6	CP085607	SRR16149125
15	13/2010	4,421,011	65.6	104 (1)	72,772	4,033	2,524,532	57.7	CP085606	SRR16149126
16	14/2010	4,417,911	64.9	127 (1)	78,950	4,005	1,949,458	44.6	CP085605	SRR16149127
17	18/2010	4,432,448	65.4	105 (1)	83,777	4,005	4,320,520	98.4	CP085603	SRR16149128
18	19/2010	4,418,613	64.9	119 (1)	88,208	4,063	2,235,076	51.1	CP085601	SRR16149130
19	20/2010	4,417,343	65.3	122 (1)	70,648	4,072	2,072,368	70.8	CP085599	SRR16149134
20	21/2010	4,417,666	65.0	103 (1)	74,425	4,057	1,931,004	44.1	CP085598	SRR16149135
21	22/2010	4,420,383	65.0	103 (1)	82,311	3,973	2,266,198	50.9	CP085596	SRR16149136
22	23/2010	4,420,918	65.1	107 (1)	82,358	4,053	2,646,318	60.5	CP085595	SRR16149157
23	24/2010	4,420,288	65.0	117 (1)	89,830	4,029	2,693,386	61.6	CP085594	SRR16149139
24	38/2010	4,419,478	65.1	118 (1)	82,355	4,041	2,480,456	56.7	CP085591	SRR16149142
25	39/2010	4,419,821	65.0	113 (1)	70,550	3,996	2,645,606	60.5	CP085590	SRR16149144
26	41/2010	4,419,996	65.2	124 (1)	81,940	3,969	2,478,720	56.7	CP085589	SRR16149145
27	45/2010	4,423,281	64.9	126 (1)	83,828	4,023	3,100,236	70.8	CP085588	SRR16149146
28	49/2010	4,419,930	65.1	108 (1)	86,634	4,025	1,673,088	38.2	CP085587	SRR16149147
29	52/2010	4,420,722	64.7	112 (1)	97,511	4,031	1,889,694	43.2	CP085586	SRR16149148
30	60/2010	4,419,012	65.0	112 (1)	74,504	4,026	1,640,690	37.5	CP085585	SRR16149149
31	62/2010	4,420,147	64.9	120 (1)	75,270	4,026	2,078,860	47.5	CP085584	SRR16149150
32	72/2010	4,418,098	65.0	100 (1)	79,076	4,013	1,681,112	38.4	CP085583	SRR16149151
33	32/2011	4,425,788	64.9	254 (1)	58,890	4,072	1,354,132	46.3	CP085582	SRR16149153
34	78/2011	4,417,082	65.4	254 (1)	58,890	4,034	604,650	20.7	CP086331	SRR16149152
35	169/2011	4,435,970	64.7	116 (1)	122,223	4,041	7,912,402	180.1	CP085573	SRR16149163
36	397/2013	4,439,340	64.7	128 (1)	140,939	4,067	10,194,418	231.9	CP085572	SRR16149164
37	728/2015	4,440,561	64.9	170 (1)	45,940	4,040	11,277,128	256.5	CP085581	SRR16149155
38	729/2015	4,431,948	65.1	137 (1)	122,217	4,063	7,806,876	177.9	CP085580	SRR16149156
39	23/2016	4,433,118	65.1	122 (1)	101,672	3,983	8,481,276	190.2	CP085579	SRR16149138
40	25/2016	4,434,896	65.0	124 (1)	88,942	4,067	8,583,572	195.5	CP085578	SRR16149158
41	56/2016	4,427,560	65.0	139 (1)	98,673	4,066	7,818,980	178.4	CP085577	SRR16149159
42	129/2016	4,430,911	65.1	134 (1)	103,078	4,062	8,574,282	195.4	CP085576	SRR16149160
43	539/2016	4,440,780	64.8	119 (1)	105,756	4,053	9,853,192	224.1	CP085571	SRR16149166
44	541/2016	4,429,660	65.0	109 (1)	128,835	4,056	9,203,682	209.9	CP085575	SRR16149161
45	734/2016	4,427,845	64.9	132 (1)	107,136	4,048	7,669,828	175.0	CP085574	SRR16149162
46	800/2016	4,440,527	64.5	123 (1)	114,329	4,059	11,146,772	253.6	CP085570	SRR16149167
47	275/2017	4,419,700	64.9	163 (1)	59,075	4,033	3,362,504	114.9	CP085564	SRR16149173
48	296/2017	4,418,199	65.0	155 (1)	59,898	4,048	2,407,596	82.3	CP085566	SRR16149171
49	297/2017	4,418,725	64.9	149 (1)	59,360	4,018	2,221,230	75.9	CP085563	SRR16149174
50	520/2017	4,418,006	65.0	165 (1)	52,153	4,063	2,215,626	75.7	CP085569	SRR16149168
51	525/2017	4,418,536	64.9	165 (1)	57,285	4,070	2,542,826	86.9	CP085565	SRR16149172
52	715/2017	4,420,895	65.1	255 (1)	59,214	4,059	2,583,466	88.2	CP085547	SRR16149143
53	731/2017	4,419,645	65.2	157 (1)	53,863	4,058	2,354,200	80.4	CP085548	SRR16149132
54	1207/2017	4,414,348	65.1	152 (1)	47,575	4,067	1,472,358	50.4	CP085542	SRR16149188
55	1361/2017	4,416,102	65.0	172 (1)	56,637	4,053	2,488,510	85.1	CP085549	SRR16149121
56	1400/2017	4,414,254	65.2	167 (1)	53,201	4,050	1,770,318	60.6	CP085550	SRR16149113
57	1408/2017	4,414,653	65.1	166 (1)	57,730	4,061	1,759,172	60.2	CP085567	SRR16149170
58	300/2017	4,415,628	65.0	152 (1)	52,153	4,064	1,202,309	41.1	CP085568	SRR16149169
59	392/2018	4,418,914	64.9	152 (1)	74,473	4,054	2,777,068	94.9	CP085557	SRR16149181
60	405/2018	4,418,415	64.9	145 (1)	59,406	4,070	2,331,796	79.7	CP085551	SRR16149112
61	416/2018	4,418,171	64.9	172 (1)	55,994	4,075	1,995,066	68.2	CP085559	SRR16149179
62	425/2018	4,418,046	64.9	146 (1)	66,999	4,058	2,593,182	88.7	CP085558	SRR16149180
63	431/2018	4,420,078	64.9	144 (1)	57,124	4,057	2,570,998	87.8	CP085546	SRR16149154
64	525/2018	4,417,688	65.0	170 (1)	51,868	4,052	2,274,844	77.8	CP085562	SRR16149175
65	537/2018	4,417,657	64.8	161 (1)	57,388	4,080	2,128,110	72.7	CP085556	SRR16149182
66	543/2018	4,419,294	64.8	150 (1)	56,146	4,080	2,194,518	75.0	CP085554	SRR16149184
67	545/2018	4,419,615	64.8	151 (1)	64,246	4,082	2,528,728	86.4	CP085552	SRR16149186
68	816/2018	4,417,436	65.0	146 (1)	61,065	4,067	2,191,714	74.9	CP085561	SRR16149177
69	993/2018	4,415,299	65.0	152 (1)	58,168	4,042	2,494,242	85.3	CP085560	SRR16149178
70	1072/2018	4,415,762	65.0	149 (1)	56,052	4,065	2,358,978	80.7	CP085555	SRR16149183
71	1168/2018	4,414,894	65.0	147 (1)	62,519	4,039	1,863,456	63.7	CP085553	SRR16149185
72	253/2019	4,476,622	65.2	112 (1)	98,635	4,056	3,753,884	125.8	CP085615	SRR16149116
73	270/2019	4,420,676	65.1	150 (1)	56,699	4,050	2,865,294	97.9	CP085545	SRR16149165
74	293/2019	4,447,276	66.0	111 (1)	88,142	4,060	3,180,676	107.3	CP085616	SRR16149115
75	546/2019	4,444,377	66.0	96 (1)	83,190	4,048	2,837,276	95.8	CP085617	SRR16149114
76	598/2019	4,420,900	65.1	147 (1)	73,760	4,061	2,948,322	100.7	CP085544	SRR16149176
77	952/2019	4,420,797	65.0	148 (1)	64,230	4,021	3,012,618	102.9	CP085543	SRR16149187

a CDS, coding DNA sequences.

## References

[1] Panaiotov S, Madzharov D, Hodzhev Y. 2022. Biodiversity of *Mycobacterium tuberculosis* in Bulgaria related to human migrations or ecological adaptation. Microorganisms 10:146. doi:10.3390/microorganisms10010146. 35056596 PMC8778017

[2] Panaiotov S, Bachiyska E, Yordanova S, Atanasova Y, Todorova Y, Levterova V, Simeonovski I, Tankova K, Stefanova T, Karcheva A, Kantardjiev T. 2016. Genetic biodiversity of sensitive and multi-resistant strains of *Mycobacterium tuberculosis* in Bulgaria. (in Bulgarian). Meditsinski Pregled (Medical Rev) 52:47–54.

[3] Yordanova S, Baykova A, Atanasova Y, Todorova Y, Bachiyska E. 2020. Isoniazid-monoresistant tuberculosis in Bulgaria. Probl Inf Parasit Dis 48:21–24. https://pipd.ncipd.org/index.php/pipd/article/view/29.

[4] Yordanova S, Bachiyska E, Atanasova Y, Todorova Y, Baykova A, Kantardjiev T. 2013. Multidrug resistant tuberculosis in Bulgaria: microbiological aspects. Probl Inf Parasit Dis 41:5–8.

[5] Kamerbeek J, Schouls L, Kolk A, van Agterveld M, van Soolingen D, Kuijper S, Bunschoten A, Molhuizen H, Shaw R, Goyal M, van Embden J. 1997. Simultaneous detection and strain differentiation of *Mycobacterium tuberculosis* for diagnosis and epidemiology. J Clin Microbiol 35:907–914. doi:10.1128/jcm.35.4.907-914.1997. 9157152 PMC229700

[6] Supply P, Allix C, Lesjean S, Cardoso-Oelemann M, Rüsch-Gerdes S, Willery E, Savine E, de Haas P, van Deutekom H, Roring S, Bifani P, Kurepina N, Kreiswirth B, Sola C, Rastogi N, Vatin V, Gutierrez MC, Fauville M, Niemann S, Skuce R, Kremer K, Locht C, van Soolingen D. 2006. Proposal for standardization of optimized Mycobacterial Interspersed Repetitive Unit-Variable Number Tandem Repeat typing of *Mycobacterium tuberculosis*. J Clin Microbiol 44:4498–4510. doi:10.1128/JCM.01392-06. 17005759 PMC1698431

[7] Van Soolingen D, de Haas PE, Hermans PW, van Embden JD. 1994. DNA fingerprinting of *Mycobacterium tuberculosis*. Methods Enzymol 235:196–205. doi:10.1016/0076-6879(94)35141-4. 8057895

[8] Schmieder R, Edwards R. 2011. Quality control and preprocessing of metagenomic datasets. Bioinformatics 27:863–864. doi:10.1093/bioinformatics/btr026. 21278185 PMC3051327

[9] Magoc T, Salzberg SL. 2011. FLASH: fast length adjustment of short reads to improve genome assemblies. Bioinformatics 27:2957–2963. doi:10.1093/bioinformatics/btr507. 21903629 PMC3198573

[10] Kearse M, Moir R, Wilson A, Stones-Havas S, Cheung M, Sturrock S, Buxton S, Cooper A, Markowitz S, Duran C, Thierer T, Ashton B, Meintjes P, Drummond A. 2012. Geneious Basic: an integrated and extendable desktop software platform for the organization and analysis of sequence data. Bioinformatics 28:1647–1649. doi:10.1093/bioinformatics/bts199. 22543367 PMC3371832

[11] Tatusova T, DiCuccio M, Badretdin A, Chetvernin V, Nawrocki EP, Zaslavsky L, Lomsadze A, Pruitt KD, Borodovsky M, Ostell J. 2016. NCBI Prokaryotic Genome Annotation Pipeline. Nucleic Acids Res 44:6614–6624. doi:10.1093/nar/gkw569. 27342282 PMC5001611

